# Complete plastome sequence of *Osmanthus armatus* and phylogenetic implications in Oleaceae

**DOI:** 10.1080/23802359.2019.1666686

**Published:** 2019-09-19

**Authors:** Yingxue Du, Rui Li, Qingde Zhang

**Affiliations:** a Key Laboratory of Plant Stress Biology, School of Life Sciences, Henan University, Kaifeng, China;; b Physical and Chemical Laboratory, Food inspection and Testing Institute of Henan Province, Zhengzhou, China

**Keywords:** *Osmanthus armatus*, plastome, phylogeny inference

## Abstract

*Osmanthus armatus* (Oleaceae) is mainly distributed in the southwest of China and also planted in arboretums as an ornamental plant. In the present study, the plastome of *O. armatus* was reconstructed using genome skimming sequencing, and the phylogeny analysis was inferred based on whole plastome data. The plastome of *O. armatus* is 155,259 bp in length, comprising two copies of inverted regions (IR, 25,680 bp) separated by the large single copy (LSC, 86,534 bp) and small single copy (SSC, 17,365 bp) regions. The genome encodes 114 unique genes, including 80 different protein-coding genes, 30 tRNA genes, and four rRNA genes, with 20 duplicated genes in the IR regions. Phylogenetic analysis suggests that the representative species from *Osmanthus* is monophyletic, and *O. armatus* is sister to *O. fragrans* within this genus.

The genus *Osmanthus* includes about 30 fragrant species of small trees and shrubs from northern American, Eastern Europe, South East Asia, and the Pacific Islands (Wu and Raven 2001). *Osmanthus armatus* Diel., which is a member of this genus, is mainly distributed in the southeast of China and also planted in arboretums as an ornamental plant (Yin et al. 2010). In addition, this species is usually used for clearing heat and detoxifying, promoting blood circulation and relieving pain in China. However, currently there is no information available regarding its genetic background. In this study, the plastome of *O. armatus* was determined using genome skimming data. This genome sequence was deposited into GenBank (accession number: MN207124).

The whole genomic DNA was extracted from silica-dried leaf tissue of one *O. armatus* individual collected in Wuhan Botanical Garden (China; 114°25′09.49″E, 30°32′41.34″N) using modified CTAB reagent (Plant DNAzol, Shanghai, China) according to the manufacturer’s protocol. A voucher specimen (*Luxian Liu LLX18112401*) was deposited at the Herbarium of Henan University (HHN). High-quality DNA was sheared and the 500 bp short-insert length paired-end library was sequenced in one lane of an Illumina Hiseq X10 by Beijing Genomics Institute (BGI, China) and obtained reads with length of 150 bp. The raw reads were screened by quality with Phred score < 30, and all the remaining reads were assembled into contigs implemented in CLC genome workbench (CLC Inc., Rarhus, Denmark) to reconstruct the plastome with *O. insularis* (GenBank: MH817862) as a reference. The software Geneious R11 (Biomatters, Auckland, New Zealand) was used to annotate the plastome of *O. insularis* as described in Liu et al. (2017), and the circular plastome map was drawn utilizing the OrganellarGenomeDRAW tool (OGDRAW). Phylogeny relationships of Oleaceae were inferred using the whole plastome sequences, and maximum-likelihood (ML) was implemented in RAxML-HPC v8.1.11 on the CIPRES cluster (Miller et al. 2010).

The plastome of *O. armatus* is 155,259 bp and had a typical quadripartite structure with an 86,534 bp large single copy region (LSC), a 17,365 bp small single copy region (SSC) and two 25,680 bp inverted repeats. The plastome contains 114 unique genes including 80 protein-coding genes, 30 tRNA genes, and four ribosomal RNA genes, with 20 genes duplicated in the IR regions. Nine protein-coding genes and six tRNA genes contain one intron, as well as three protein-coding genes (*clp*P, *ycf*3, and *rps*12) contain two introns. The constructed phylogeny indicated that five representative species from *Osmanthus*, including *O. fragrans*, *O. armatus, O. insularis, O. yunnanensis*, and *O. delavayi*, formed a highly supported clade, and *O. armatus* is sister to *O. fragrans* within this genus (Figure 1).

**Figure 1.**
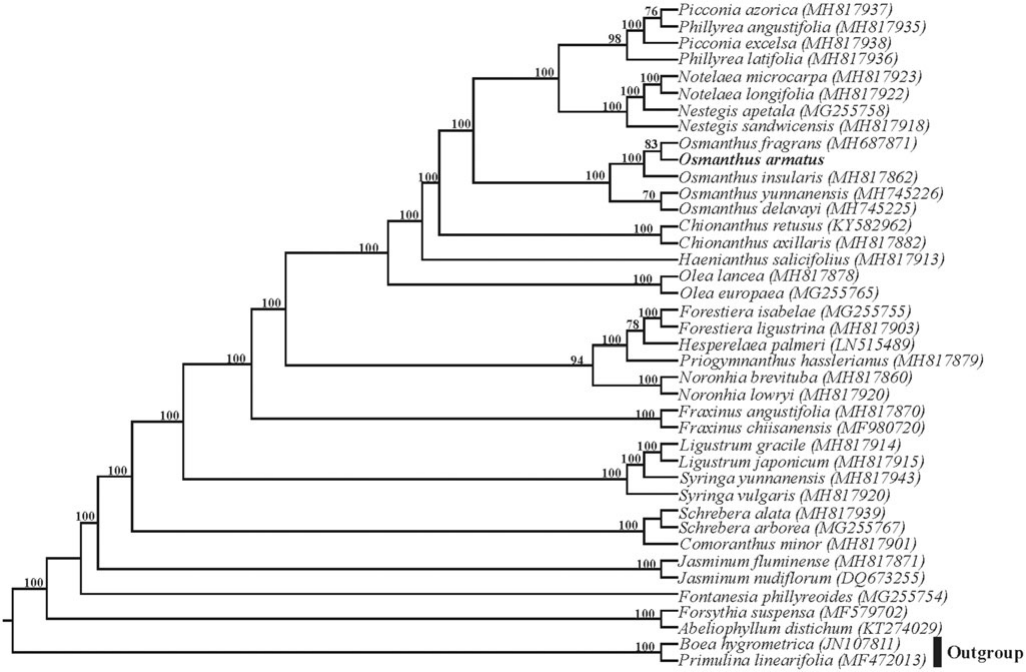
Phylogenetic relationships of Oleaceae inferred based on whole cp genome sequences. Numbers above the branches represent bootstrap values from maximum likelihood analyses. Support values of less than 50% are not shown. GenBank accession numbers of taxa are shown after the species name.

In conclusion, the plastome of *O. armatus* is reported for the first time in this study. It will provide essential and important genetic resources for future better development and utilization of this fragrant species, as well as give insights into evolutionary relationships within the Oleaceae family.
